# Uterine NK Cells Are Critical in Shaping DC Immunogenic Functions Compatible with Pregnancy Progression

**DOI:** 10.1371/journal.pone.0046755

**Published:** 2012-10-08

**Authors:** Irene Tirado González, Gabriela Barrientos, Nancy Freitag, Teresa Otto, Victor L. J. L. Thijssen, Petra Moschansky, Petra von Kwiatkowski, Burghard F. Klapp, Elke Winterhager, Stefan Bauersachs, Sandra M. Blois

**Affiliations:** 1 Medicine University of Berlin, Charité Centre 12 Internal Medicine and Dermatology, Laboratory of Reproductive Medicine, Berlin, Germany; 2 Laboratorio de Fisiología Molecular Placentaria, Departamento de Química Biológica, Facultad de Ciencias Exactas y Naturales, Universidad de Buenos Aires, Buenos Aires, Argentina; 3 Institute of Molecular Biology, University Hospital, University Duisburg-Essen, Essen, Germany; 4 Department of Radiotherapy, Angiogenesis Laboratory, VU University Medical Center, Amsterdam, The Netherlands; 5 Chair for Molecular Animal Breeding and Biotechnology, Gene Center, LMU Munich, Munich, Germany; Institute of Zoology, Chinese Academy of Sciences, China

## Abstract

Dendritic cell (DC) and natural killer (NK) cell interactions are important for the regulation of innate and adaptive immunity, but their relevance during early pregnancy remains elusive. Using two different strategies to manipulate the frequency of NK cells and DC during gestation, we investigated their relative impact on the decidualization process and on angiogenic responses that characterize murine implantation. Manipulation of the frequency of NK cells, DC or both lead to a defective decidual response characterized by decreased proliferation and differentiation of stromal cells. Whereas no detrimental effects were evident upon expansion of DC, NK cell ablation in such expanded DC mice severely compromised decidual development and led to early pregnancy loss. Pregnancy failure in these mice was associated with an unbalanced production of anti-angiogenic signals and most notably, with increased expression of genes related to inflammation and immunogenic activation of DC. Thus, NK cells appear to play an important role counteracting potential anomalies raised by DC expansion and overactivity in the decidua, becoming critical for normal pregnancy progression.

## Introduction

The early events taking place at the mouse and human endometrium following implantation determine the most critical period for successful mammalian pregnancy. During early stages, stromal cell proliferation and differentiation must be properly coordinated with the angiogenic development of the uterine vascular bed to support decidual development. Abnormalities during this period are often linked to complications such as preeclampsia, intrauterine growth restriction and premature pregnancy termination, which have a strong impact on offspring health [1].

Many of the early signals involved in pregnancy maintenance are derived from immune cell populations that infiltrate the decidual tissue, the most abundant being NK cells. These unique cells are massively recruited to the implantation site during decidualization in mice and have for long been recognized as important regulators of spiral artery remodeling and the maintenance of decidual integrity [2]–[4]. It was recently acknowledged that the normal recruitment and functional properties of uterine NK (uNK) cells are partially dependent on signals derived from DC, which dramatically increase their numbers at the onset of implantation and persist in the uterus throughout mouse gestation [5], [6]. Indeed, DC depleted implantation sites are characterized by decreased levels of IL-15 resulting in reduced numbers and impaired differentiation of NK cells [7], which thus fail to produce normal levels of IFN-γ necessary for spiral artery remodeling [8]. More recently, the finding that DC depletion provokes implantation failure in mice due to impaired decidua formation and vascularization has led to the assumption that these cells are the most prominent subset to determine the outcome of pregnancy [6]. Yet, in view of the importance of DC derived signals for the normal functions of the NK cell pool, the severe defects associated with DC depletion may also reflect a disruption of cooperative effects mediated by both cell subsets. This is consistent with findings from *in vitro* studies showing that trophoblasts fail to induce a proliferative response in uterine cell cultures depleted of DC and NK cells [9].

A cooperative dialogue between DC and NK cells, in which they help each other to become fully mature and functional, modulates innate and adaptive immune responses against tumors and infections [10], [11]. The intimate cell-cell contact required for such DC-NK cell cross-talk is also observed in decidual tissue during pregnancy in mice and humans [9], [12], and there is indeed evidence from *in vitro* human studies showing enhanced NK cell proliferation and activation upon co-culture with decidual DC [13]. It has also been reported that human DC improve their capacity to induce regulatory T cells upon interaction with uNK cells [14] and that reciprocally, tolerogenic uterine DC promote the proliferation and differentiation of IL-10 producing NK cells [15]. Thus, this cross-talk may be important to restrain immunogenic activation of DC and NK cells in the uterus, keeping their functions compatible with successful pregnancy. However, the impact of DC-NK cell interactions on regulatory mechanisms promoting the maintenance of pregnancy *in vivo* has not been investigated. With the aim of identifying interactions between these subsets potentially involved in the orchestration of endometrial changes during early pregnancy, we analysed the effect of manipulating the relative abundance of DC and NK cells in the mouse uterus at the onset of implantation.

## Materials and Methods

### Animals

Five- to six-week C57BL/6 CD11c.DTR female mice, which express the diphteria toxin receptor (DTR) under the control of the CD11c promoter as described by Plaks *et al.* [16], were purchased from Jaxmice® and maintained in our animal facility with a 12L/12D cycle. The presence of a vaginal plug after cohabitation of CD11c.DTR females with Balb/c males was denoted as gestation day (gd) 0.5.

### Conditional ablation of DC and NK cells

CD11c.DTR females with vaginal plugs were separated from males and injected i.p. on gd 4.5 with either diphtheria toxin (DT; Sigma-Aldrich, 2 ng/g body weight in phosphate buffered saline (PBS) supplemented with rabbit normal serum) or rabbit anti-asialo GM1 antibody (Ab) (WAKO, Cat 986-10001, 2 µg/g BW) for depletion of DC and NK cells respectively. The combined depletion of both cell subsets was achieved by injecting a cocktail of DT and the anti-asialo GM1 Ab at the same concentrations. Control CD11c.DTR females received i.p. injection of PBS supplemented with rabbit normal serum (2 µg/g body weight). Procedures that involved mice were approved by LaGeSo (licence G0280/10) state authority for Animal Use in Research and Education and were conducted in strict accordance with guidelines for the care and use of laboratory research animals promulgated by the Medicine University of Berlin. On gd 5.5, 6.5 and 7.5, mice from the respective groups (n = 6–7) were sacrificed and uterine tissue from the implantation sites was processed for histological sectioning and isolation of total protein and RNA according to standard procedures.

### Fms-related tyrosine kinase 3 ligand (FL) treatment

In order to expand uterine DC during early pregnancies, some Balb/c mated CD11c.DTR females were treated with one daily i.p. injection of human recombinant FL (BioX cell, Cat No. BE0098, 10 µg/mouse/day) starting on the morning of vaginal plug detection (gd 0.5). Treatment continued for 5, 6 or 7 consecutive days, depending of the experimental design. The combined DC expansion and NK cell depletion was achieved in such FL treated female mice by replacing the injection on gd 4.5 with a cocktail of FL+anti-asialo GM1 Ab at the same concentrations as described above.

### Flow Cytometric Analysis

Isolation of uterine cells for flow cytometric analysis was carried out as previously described [17]. Expression of cell surface antigens was evaluated by direct immunofluorescence, except DC, NK lectin group receptor-1 (DNGR-1); which required indirect immunofluorescence. Approximately 5×10^5^ cells per sample were incubated with mAb for 30 min at 4°C, washed with Fluorescence activated cell sorting (FACS) buffer (PBS supplemented with 1% Bovine serum albumin (BSA) and 0.1% sodium azide). The following mAbs were used from BD Pharmingen: cluster of differentiation (CD)11c-FITC, CD4-PerCP, CD8-APC, CD80-PE, Major histocompatibility complex (MHC) class II-PE. Mouse plasmacytoid dendritic cell antigen-1 (PDCA-1)-APC was purchased from Miltenyi Biotec. DNGR-1 expression was determined by using biotinylated anti-mouse DNGR-1 (a kind gift from Dr. Caetano Reis e Sosa, Cancer Research UK, London Research Institute), followed by a secondary with streptavidin from BD Pharmingen. The acquisition (100,000 events) was performed using a FACScalibur analyzer (Becton Dickinson). Flow cytometry compensation was set in each experiment using single-color stained samples. FlowJo software was used for data analysis. Flow cytometry results were expressed as the percentage of cells positive for the surface marker evaluated.

### Immunohistochemistry

Serial sections from multiple implantation sites (2–3) and females (4–7) were stained at gd 5.5 and 6.5 after our standard protocol [6]. Detailed descriptions of this protocol are available as Text S1.

### Soluble fms-related tyrosine kinase 1 (Flt-1) ELISA

The quantification of Flt-1 serum levels was performed using the mouse Flt-1 Quantikine Immunoassay (R&D Systems, Cat MVR100) following the manufacturer's recommendations (please refer to Text S1).

### Quantitative Reverse Transcriptase – Polymerase Chain Reaction (qRT-PCR)

Total RNA isolation, subsequent cDNA synthesis, and real time PCR were performed as described [17]. Primer sequences are detailed in Table S1.

### Gene expression profiling using Affymetrix microarrays

For microarray analysis, the GeneChip® Gene 1.0 ST Array System was used (Affymetrix UK Ltd, High Wycombe, UK). Biotinylated cDNA was produced starting from 250 ng total RNA with the GeneChip® WT Sense Target Labeling and Control Reagents kit (Affymetrix, order no. 900652) and hybridized to GeneChip® Mouse Gene 1.0 ST Arrays (Affymetrix, order no. 901169) according to the manufacturer's instructions. Hybridized arrays were processed with the GeneChip® Fluidics Station 450 (Affymetrix) and scanned with the GeneChip® Scanner 3000 7G (Affymetrix). Affymetrix Expression Console™ Software (version 1.1) was used to calculate gene level RMA-processed signal intensities. Data above background information was used to filter out probe sets that were not detectable to reduce background noise. Significance analysis was performed using the Microsoft Excel add-in ‘Significance analysis of microarrays’ (SAM, two-class unpaired) [18]. For Gene Set Enrichment Analysis (GSEA) [19], all expressed genes were pre-ranked based on expression fold change and SAM q-value (log2(fold change+2)*-log10(q-value)). This pre-ranked gene list was compared with GSEA gene sets c2.all.v3.symbols.gmt (curated) and other published gene sets (sets of differentially expressed genes in other studies).

### Statistics

The number of animals included in each experimental group was indicated accordingly. Data are presented as mean ± SD from three replicate experiments. Statistical significance was determined using analysis of variance and Tukey's test, with a P value of less than 0.05 being considered as significant. Statistical analysis was carried out with GraphPad Prim 6.0 (GraphPad Software Inc.).

## Results

### Combined DC and NK cell depletion severely affects decidualization leading to early pregnancy loss

**Figure 1:**
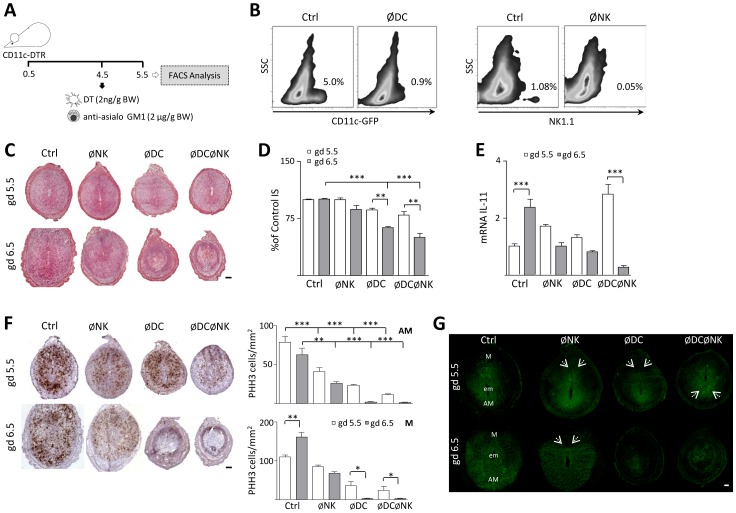
Combined depletion of DC and NK cells arrests decidual development leading to early pregnancy loss. (A) Experimental design: upon cohabitation with Balb/c males, plug-positive CD11c-DTR females were injected i.p. with either DT, anti-asialo GM1 or a combination of both for DC and/or NK cell depletion on gd 4.5, as described in Methods. Depletion of both subsets was confirmed using FACS analysis of uterine cell suspensions obtained during gd 5.5. (B) DT and anti-asialo GM1 treatment on gd 4.5 efficiently deplete DC and NK cells from the uterus. Left panels: Percentage of CD11c^+^ cells, which in CD11c-DTR mice co-express a GFP transgene, was analysed by FACS in control (Ctrl) and DT injected (ØDC) mice. Right panels: representative flow cytometric analysis of uterine cell suspensions obtained during gd 5.5 for the presence of NK cells. (C) Microscopical assessment of H&E stained serial sections revealed abnormalities in the decidual architecture of DC depleted (ØDC) and double DC-NK depleted (ØDCØNK) implantation sites (IS), with abnormal development of the antimesometrial and mesometrial compartments and signs of embryo arrest on gd 6.5. (D) Morphometric analysis of the IS diameter at gd 5.5 and 6.5. Sizes in the different groups analysed are presented as percentage of Ctrl IS. (E) Uterine IL-11 mRNA levels on gd 5.5 and 6.5, as measured by RT-PCR. The progressive increase on IL-11 expression in Ctrl females was abrogated by all treatments, with significantly reduced levels observed in ØDCØNK on gd 6.5. (F) Immunohistochemical staining of phosphorylated histone H3 (PHH3) in the mouse uterus during decidualization on gd 5.5 and 6.5. Right panel: quantification of PHH3^+^ stromal cells at the AM and M regions of the implantation sites. PHH3^+^ cells were counted per mm^2^ using magnification ×400. (G) Immunofluorescence analysis of connexin 43 (Cx-43) on gd 5.5 and 6.5. The photomicrographs of representative uterine sections are shown at 50×. Abbreviations: M = mesometrial pole, em = embryo and AM = anti-mesometrial pole of the implantation sites. Arrows indicate the regions where Cx-43 is differentially expressed in ØNK, ØDC, ØDCØNK and control implantations on gd 5.5 and 6.5. In all figures, the bars denote the means for each group in which 7 mice/group were analysed. *, ** and *** denote *p<0.05*, *p<0.01* and *p<0.001* respectively, as analysed by the Tukey's test. Scale bars: 200 µm.

DC and NK cells cooperate in the promotion of uterine cell proliferation triggered by trophoblast cells *in vitro* [9], which could have important implications for endometrial decidualization. To investigate whether this cooperation occurs *in vivo*, we aimed at comparing pregnancy progression upon combined administration of DT, which selectively depletes DC in CD11c.DTR transgenic mice, with anti-asialo GM1 antibody treatment for NK cell depletion [6], [20] at the onset of implantation (Fig. 1A). An antibody-mediated ablation strategy was chosen over a mouse strain with a genetical NK cell deficiency (e.g. *IL-15* ^−/−^ mice) because we pursued temporal ablation of NK cells during our experiment (i.e., for 72 h), avoiding complete absence of uNK cells. As shown in Figure 1B, FACS analysis on uterine cell suspensions obtained during gd 5.5 confirmed that DT and anti-asialo GM1 treatment on gd 4.5 efficiently deplete DC and NK cells respectively. The percentage of CD11c^+^ cells was approximately 4-fold decreased with respect to controls in DT-injected (ØDC) mice. In addition, treatment with anti-asialo GM1 (ØNK) resulted in a 10-fold decrease of the percentage of uterine NK cells (NK1.1^+^) compared to control mice.

Histological analysis of uterine tissue sections obtained on gd 5.5 revealed that while all groups displayed signs of embryo implantation, the size of the implantation sites observed in ØDC and ØDCØNK females were slightly decreased compared to control mice (Fig. 1C–D). As gestation progressed, on gd 6.5, these groups displayed a significant decrease of the implantation size compared to control mice (*P<0.001*, Fig. 1D), which was associated with an arrested development of the antimesometrial and mesometrial deciduas and signs of embryo resorption (Fig. 1C). In contrast, implantation sites of NK cell depleted mice were similar in size and morphology to that observed in control females.

We next analysed the expression of IL-11, which is considered a master regulator of stromal cell proliferation and differentiation during decidualization [21]. Normally, IL-11 mRNA is undetectable from gd 0.5 to 3.5, becoming expressed post-implantation and peaking between gd 5.5 and 7.5 in mice [21]. Decidual mRNA levels of IL-11 increased from gd 5.5 to 6.5 in the control mice, but this up-regulation could not be detected upon individual depletion of DC and NK cells (Fig. 1E). Interestingly, IL-11 expression on gd 5.5 was significantly increased in ØDCØNK females compared to controls (*P<0.01*, Fig. 1E), but dropped dramatically to levels comparable to those observed in ØDC and ØNK mice on gd 6.5. The immunohistochemical assessment of phosphorylated histone H3 (PHH3) further revealed that the normal dynamics of stromal cell proliferation during decidualization was disrupted upon depletion of DC and/or NK cells (Fig. 1F). In particular, significantly decreased densities of PHH3^+^ cells were observed at the antimesometrial pole of ØNK implantation sites on gd 5.5, denoting an impaired proliferation of stromal cells that further compromised both decidual compartments on gd 6.5 (Fig. 1F, right panels). Additionally, arrested decidual development in ØDC and ØDCØNK females was associated with significantly reduced amounts of proliferating cells affecting both decidual poles on gd 5.5, and a significant downregulation of the PHH3 signal from gd 5.5 to 6.5. The differentiation of stromal cells during decidualization was also severely compromised in these groups, as noted by the reduced expression of the gap junction protein connexin-43 (Cx-43) observed on gd 6.5 (Fig. 1H). In contrast, the pattern of Cx-43 staining in ØNK females was similar to control mice, though expression at the mesometrial region was decreased on gd 6.5.

### NK cells are critical for the maintenance of early pregnancy following DC expansion

**Figure 2:**
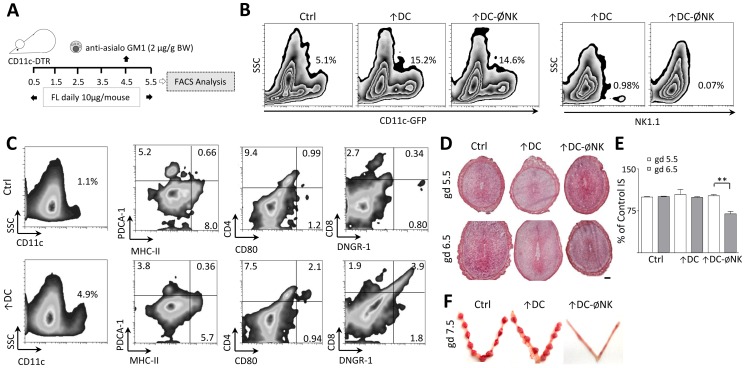
NK cells become critical for normal pregnancy progression upon DC expansion. (A) Model for DC expansion during early pregnancy. Upon plug detection; female mice were injected daily with 10 µg FL i.p. This strategy was combined with a single anti-asialo GM1 injection on gd 4.5 to analyse effects of NK depletion upon expansion of DC (↑DCØNK mice). (B) Representative flow cytometric analysis of uterine cell suspensions obtained on gd 5.5 to confirm DC expansion in the uterus upon FL treatment. FL treated (↑DC) mice exhibited percentages of CD11c^+^ cells approximately 3-fold higher than controls. (C) Phenotypic characterization of uterine DC expanded upon FL treatment. Uterine cells from ↑DC and control females were isolated on gd 4.5, and subject to FACS analysis for the expression of CD11c, PDCA-1, CD4, CD8, MHC-II, CD80, and DNGR-1. FL treatment was associated with increased percentages of CD11c^+^DNGR-1^+^ cells, whereas other markers exhibited similar expression levels with respect to control mice. Results correspond to at least three independent experiments using three to five animals/group. (D) Histological analysis of DC expanded implantation sites. While implantation was normal upon DC expansion, a regression of embryo development is observed on gd 6.5 when both strategies were combined ↑DCØNK mice. Scale bar: 200 µm. (E) Size of the IS registered in ↑DC and ↑DCØNK mice at gd 5.5 and 6.5 expressed as percentage of control. (F) Macroscopical appearance of the uterus and IS registered during gd 7.5. The pictures show the normal phenotype of control and ↑DC IS in contrast to that observed in ↑DCØNK mice, which exhibit completely resorbed embryos.

Taking in account that DC depletion exhibited the most adverse effect on early gestation, our next aim was to investigate whether the decidual growth defects observed in ØNK females could be compensated upon expansion of DC *in vivo*. For this purpose, we treated female mice during early pregnancy with FL, a cytokine that has been shown to increase the numbers of DC in several tissues [22], [23]. This strategy was combined with a single anti-asialo GM1 injection on gd 4.5 to analyse effects of NK depletion upon expansion of DC (↑DCØNK mice) (Fig. 2A). As shown in Figure 2B, a 5 day course of FL administration to pregnant female mice resulted in a 3-fold expansion of CD11c^+^ DC in the uterus compared to controls. Additionally, the percentage of NK cells in FL treated mice was significantly decreased upon treatment with anti-asialo GM1. Flow cytometric characterization of CD11c^+^ cells showed that CD4, MHC-II, CD80, CD8 and PDCA-1 expression was not altered upon FL treatment, whereas the percentage of DNGR-1^+^CD11c^+^ cells was significantly increased in ↑DC mice (Fig. 2C).

Morphological analysis of the implantation sites on gd 5.5 and 6.5 showed that expansion of DC did not alter the progression of early pregnancy, as ↑DC females displayed implantation sites with sizes comparable to those observed in control mice (Fig. 2D). In contrast, NK cell depletion following DC expansion led to a significant reduction of the implantation size compared to control mice on gd 6.5 (*P<0.05*, Fig. 2D). Defective growth of the implantation sites in ↑DCØNK mice resulted in early pregnancy failure, as evidenced by the complete resorption of embryos observed on gd 7.5 (Fig. 2F).

**Figure 3:**
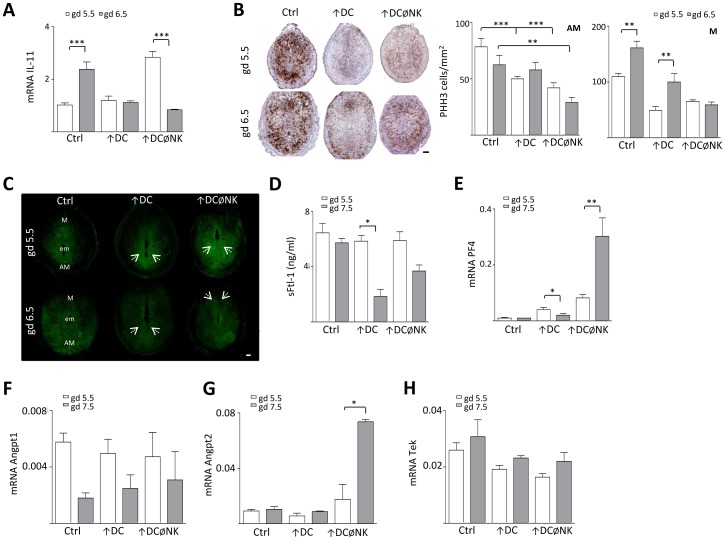
NK cell depletion following DC expansion interferes with the regulation of decidual angiogenesis. (A) IL-11 mRNA levels were analysed in uterine tissue on gd 5.5 and 6.5 by RT-PCR. Significantly reduced levels observed were observed in ↑DCØNK on gd 6.5. (B) Analysis of PHH3 staining in the mouse uterus during decidualization on gd 5.5 and 6.5. Quantification of PHH3^+^ stromal cells at the AM and M regions of the implantation sites is shown in the right panel. PHH3^+^ cells were counted per mm^2^ using magnification ×400. (C) Connexin 43 expression analysis was performed on gd 5.5 and 6.5. The photomicrographs of representative uterine sections are shown at 50×. Abbreviations: M = mesometrial pole, em = embryo and AM = anti-mesometrial pole of the implantation sites. Arrows denote differential expression of Cx-43 between ↑DC, ↑DCØNK and control implantations on gd 5.5 and 6.5. (D) Analysis of serum Flt-1 concentrations. DC expansion led to a significant decrease in sFlt-1 concentrations from gd 5.5 to gd 7.5. (E) Uterine platelet factor 4 (PF4) expression as analysed by qPCR, increased from gd 5.5 to gd 7.5 in ↑DCØNK mice, and decreased significantly upon expansion of DC. (F) Expression of Angiopoietin-1 (Angpt1) as assessed by qPCR. Decidual mRNA levels of Angpt1 in either ↑DC or ↑DCØNK females did not differ significantly from those observed in controls on gd 5.5 and gd 7.5; (G) Angiopoietin-2 (Angpt2) and (H) Tek as assessed by qPCR. No changes were induced by the treatments, except for a significant increase of Angpt2 levels in ↑DCØNK mice on gd 7.5 compared to gd 5.5. In all figures, the bars denote the means for each group in which 7 mice/group were analysed. *, ** and *** denote *p<0.05*, *p<0.01* and *p<0.001* respectively, as analysed by the Tukey's test. Scale bars: 200 µm.

We next characterized decidualization in these mice by focusing on gd 5.5 and 6.5, since signs of implantation (i.e., conceptus and embryo crypts) were still evident in all groups at these earlier stages. These experiments revealed that the normal up-regulation of decidual IL-11 expression from gd 5.5 to 6.5 was not detected in ↑DC and ↑DCØNK mice, which showed decreased mRNA levels respect to control females on gd 6.5 (Fig. 3A). Moreover, IL-11 expression in ↑DCØNK mice was significantly increased compared to controls on gd 5.5. The arrested growth of the implantation sites observed in ↑DCØNK females was related to an impaired proliferation of stromal cells in the antimesometrial and mesometrial compartments, which was detected as a significantly decreased density of PHH3^+^ cells respect to controls on gd 5.5 and 6.5 (Fig. 3B). In contrast, ↑DC females exhibited significantly decreased amounts of proliferating stromal cells on gd 5.5, but showed no differences in PHH3 expression compared to controls on gd 6.5. When analysing the Cx-43 distribution we observed that ↑DC implantations depicted a more prominent expression on the AM pole compared with control mice, but ↑DCØNK females exhibited an abnormal localization of the Cx-43 signal, which extended throughout the whole decidua on gd 5.5. On gd 6.5, decidual Cx-43 expression was less prominent in ↑DC mice compared to controls (Fig. 3C) and ↑DCØNK implantations showed a decreased Cx-43 signal in comparison to the observed on gd 5.5, suggesting that decidual differentiation is arrested in these mice.

### NK cell depletion following DC expansion up-regulates the expression of anti-angiogenic growth factors

The decidualization process occurs together with a strong angiogenic remodeling and expansion of the endometrial vascular bed, which has been shown to be critical for the maintenance of early pregnancy [24]. To investigate whether early pregnancy failure in ↑DCØNK mice may be related to an impaired decidual angiogenic response, we next analysed the expression of angiogenesis modulators upon expansion of DC. We first focused on the analysis of serum concentrations of soluble Flt-1, a trophoblast-derived molecule that negatively modulates the bioavailability of vascular endothelial growth factor (VEGF) [25]. In these experiments the analysis was conducted until gd 7.5 to encompass the whole process of decidual vascular expansion, as normally the mesometrial vascular zone is fully differentiated at this stage. As shown in Fig. 3D, serum sFlt-1 concentrations remained almost constant from gd 5.5 to gd 7.5 in control mice. In contrast, we observed a significant down-regulation of systemic sFlt-1 levels in ↑DC female mice on gd 7.5. However, upon NK cell depletion/DC expansion sFlt-1 concentrations failed to drop down as observed in ↑DC mice, suggesting that the bioavailability of VEGF may be reduced compared to DC expanded female mice.

To characterize other pathways involved in the regulation of decidual angiogenesis we next focused on platelet factor 4 (PF4), an ELR-negative chemokine that exhibits anti-angiogenic properties and is up-regulated during the course of preeclampsia [26]. Local PF4 expression in ↑DC implantation sites was low on gd 5.5, showing no significant differences respect to control females (Fig. 3E). Furthermore, PF4 expression in these mice was found to decrease as pregnancy progressed to gd 7.5. In contrast, levels of PF4 expression in ↑DCØNK implantation sites were elevated on gd 5.5, and showed a dramatical up-regulation respect to control and ↑DC mice on gd 7.5. We also analysed the angiopoietin axis, which is known to complement VEGF action during angiogenesis associated with pregnancy. In particular, angiopoietin-1 (Angpt1) is a pro-angiogenic factor that promotes vessel maturation, stabilization and leakiness through interaction with the endothelial-specific receptor tyrosine kinase Tek, while angpt2 behaves as an antagonist [27]. As depicted in Fig. 3F, decidual mRNA levels of Angpt1 in either ↑DC or ↑DCØNK females did not differ significantly from those observed in controls on gd 5.5 and gd 7.5. However, Angpt2 expression in ↑DCØNK female mice was significantly up-regulated as gestation progressed; showing increased levels respect to control and ↑DC mice on gd 7.5 (Fig. 3G). Expression levels of Tek were similar in all groups on both gestation days analysed (Fig. 3H).

### NK cell depletion following DC expansion is associated with increased expression of genes involved in immune activation and inflammation

**Figure 4:**
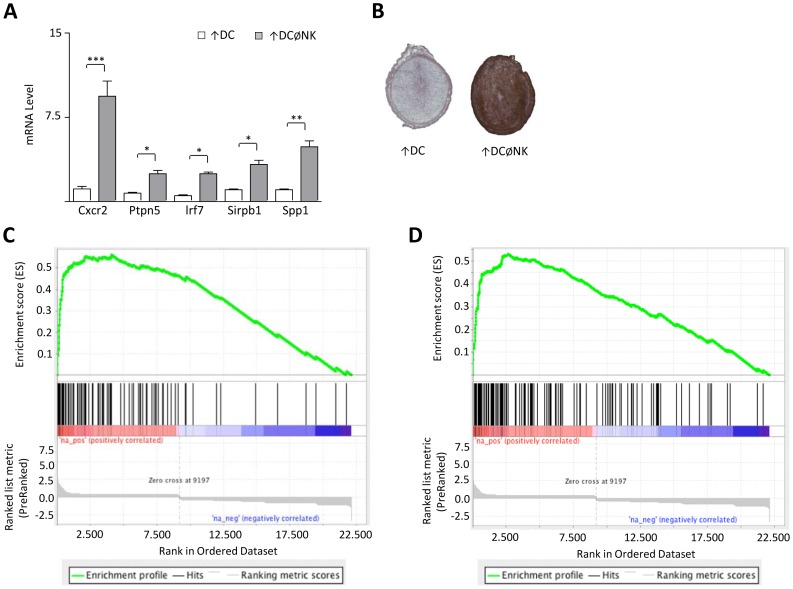
NK cells regulate DC function during early gestation. (A) Confirmation of differential gene expression on ↑DCØNK implantation sites. The pictures display qPCR results confirming up-regulated expression of chemokine (C-X-C motif) receptor 2 (Cxcr2), protein tyrosine phosphatase, non-receptor type 5 (Ptpn5), Interferon regulatory factor 7 (Irf7), signal-regulatory protein beta 1A and B (Sirpb1), and secreted phosphoprotein 1 (Spp1), as was noted on microarray analysis comparing ↑DC *vs* ↑DCØNK transcription profiles on gd 5.5. In all figures, *, ** and *** denote *p<0.05*, *p<0.01* and *p<0.001* respectively, as analysed by Mann–Whitney rank-sum test. (B) Up-regulation of progesterone receptor (Pgr) was analysed on IHC staining of uterine sections obtained during gd 5.5. (C–D) Selected enrichment plot of Gene Set Enrichment Analysis (GSEA). All detectable genes in the microarray analysis were ranked according to fold change and adjusted p-value (see Material & Methods) and compared to sets of differentially expressed genes (DEGs) from other study as well as gene sets from the GSEA database. Selected enrichment plot for gene sets enriched towards the genes up-regulated in ↑DCØNK are shown. X-axis represents the ranked data set with the most significantly up-regulated genes on the left. Black vertical bars represent the positions of the genes of the compared gene sets. The references for the gene sets can be found in the text.

To identify additional signals potentially involved in pregnancy failure triggered by NK cell depletion in FL treated mice, a microarray analysis of implantation sites collected on gd 5.5 from the ↑DC and ↑DCØNK groups was performed. Day 5.5 of pregnancy was chosen because by this day histological analysis between the ↑DC and ↑DCØNK implantation sites showed fewer differences than when compared on gd 6.5. Statistical analysis at a false discovery rate (FDR) of 10% revealed 173 genes with higher mRNA levels in ↑DCØNK samples and only 2 genes with increased expression in ↑DC samples (Table S2). Table 1 shows selected examples of differentially expressed genes found in ↑DCØNK implantation sites. Most of the up-regulated genes encode proteins well known to be involved in immune responses and immune functions such as activation and proliferation. As an example, Figure 4A shows qPCR results confirming up-regulation of Cxcr2, Ptpn5, Irf7, Sirpb1, and Spp1 in ↑DCØNK samples. Figure 4B depicts differential progesterone receptor (Pgr) expression between ↑DC and ↑DCØNK mice analysed by IHC. To further characterize the obtained gene expression data, a Gene Set Enrichment Analysis (GSEA) was performed to compare this data set to possibly related published gene sets (Table S3) [19]. Enrichment towards the genes up-regulated in ↑DCØNK mice was found for a number of gene sets in the GSEA database, in particular for genes reported as up-regulated during dendritic cell maturation [28] (Fig. 4C) and in a mouse model of acute graft vs. host disease [29] (Fig. 4D; Table S3).

**Table 1:** Summary of the results obtained in microarray experiments comparing the transcription profile of ↑DC and ↑DCØNK implantation sites obtained during gd 5.5.

Symbol	mRNA Accession No.	Name	Fold Change	q value	Function (GO)
Alox15	NM_009660	arachidonate 15-lipoxygenase	4.2	<0.001	Inflammatory response, lipoxygenase pathway
Cxcl10	NM_021274	chemokine (C-X-C motif) ligand 10	1.5	0.0319	Immune response, inflammatory response
Cdkn1a	NM_007669	cyclin-dependent kinase inhibitor 1A (P21)	1.5	0.0845	Cell cycle arrest
Pgr	NM_008829	Progesterone receptor	1.6	<0.001	Steroid hormone signalling pathway
Clec7a	NM_020008	C-type lectin domain family 7, member a	1.6	0.0845	Innate immune response, inflammatory response
Ggt5	NM_011820	gamma-glutamyltransferase 5	1.8	<0.001	Inflammatory response
Ifit1	NM_008331	interferon-induced protein with tetratricopeptide repeats 1	1.9	0.0845	Cellular response to type I IFN
Csf3r	NM_007782	colony stimulating factor 3 receptor (granulocyte)	2.1	0.0931	Cytokine signaling pathway, Neutrophil chemotaxis
Lrg1	NM_029796	leucine-rich alpha-2-glycoprotein 1	2.1	<0.001	Implantation, decidualization.
Gadd45g	NM_011817	growth arrest and DNA-damage-inducible 45 gamma	2.3	0.0845	Cell cycle regulation, activation of MAPKK activity, IFNg biosynthesis
P2ry14	NM_133200	purinergic receptor P2Y, G-protein coupled, 14	2.3	0.0507	Immune response, signal transduction
Slfn1	NM_011407	schlafen 1	2.6	<0.001	Cell cycle arrest, G1/S transition of mitotic cell cycle
Spp1	NM_009263	Secreted phosphoprotein 1	4.2	0.0212	Cytokine activity, response to steroid hormone stimulus
Sirpb1	NM_001173460	signal-regulatory protein beta 1B	1.8	<0.001	Signal transduction, positive regulation of phagocytosis
Cd80	NM_009855	Cd80 antigen	1.4	0.0318	T cell costimulation
Frem1	NM_177863	Fras1 related extracellular matrix protein 1	−2.0	0.0225	Cell communication, cell-matrix adhesion
Dgkb	NM_178681	diacylglycerol kinase, beta	−1.8	0.0225	Intracellular signal transduction

Differential expression with respect to control (↑DC) mice is expressed as fold change in mRNA levels. Putative functions associated to each gene are indicated based on the Gene Ontology project database (http://www.geneontology.org/). The complete list of differentially expressed genes (FDR 10%) can be found in Table S2.

## Discussion

Interactions between DC and NK cells are known to modulate innate and adaptive immune responses, and may be important to determine the normal functions of these cells in the uterus during early stages of pregnancy [9], [30]. In the present study, we provide novel insights about the regulatory role played by NK cells in preventing potential anomalies due to excessive DC activation during early pregnancy.

Our study first showed that unlike NK cell depleted mice, in which pregnancy progression was not overtly compromised, ØNKØDC females exhibited severe defects in decidual development resembling the early pregnancy failure phenotype previously described upon DC depletion [6]. These results are consistent with the pivotal role ascribed to DC in the control of decidual development and argue against a major physiological contribution from NK cells, in agreement with previous studies reporting normal pregnancy rates and a lack of differential expression of decidualization-related genes on gd 7.5 in IL-15 deficient mice [31], [32]. Still, the finding that the proliferation of stromal cells was negatively affected upon NK cell depletion confirms that signals derived from these cells influence the decidualization process, and provides an explanation for the abrogated proliferative response of uterine cells observed previously in NK cell depleted cultures [9] as well as the decidual hypoplasia reported in mouse strains lacking uNK cells [2]. Interestingly, our experiments also suggest that impaired proliferation and differentiation of stromal cells may result from defective levels of IL-11 expression upon depletion of DC and NK cells, which were most prominent in ØNKØDC implantation sites. In mice, IL-11 mRNA is detected mostly on cells of the antimesometrial and secondary decidua in a pattern partly overlapping the expression of its receptor [33], suggesting that decidualization is controlled by an autocrine mechanism of IL-11 signaling. While the local molecular pathways involved in the regulation of IL-11 expression are still largely elusive, our results suggest that the actions of DC and NK cells during decidualization may be related to the modulation of IL-11 synthesis by decidual cells.

A striking finding in this study was that not only were stromal proliferation defects still evident upon expansion of DC, but also that decidual growth was severely compromised in ↑DCØNK females leading to early pregnancy failure. Thus, NK cells appear to play an important regulatory role that becomes critical for normal pregnancy maintenance following expansion of DC. A hallmark of normal mouse pregnancy is a progressive decline of the tissue density of DC in the decidua over the first half of post-implantation development [34], and it has been speculated that such a mechanism would act to prevent local events of inflammation that may compromise placental development or function due to overactivity of decidual DC. Indeed, our results showed that FL- triggered DC expansion was associated with an increased frequency of DNGR-1-expressing CD11c^+^ cells, which have been shown to elicit robust cross-priming responses involved in antitumor immunity [35]. Yet, increased tissue densities of such an immunogenic DC subset were not detrimental for pregnancy *per se*, but only when the dynamics of NK cell recruitment to the uterus was disrupted. In this context, the proven influence of uterine DC on pathways promoting the recruitment and differentiation of NK cells during early pregnancy [7], [8] would represent a self-directed physiological mechanism aimed at restraining the immunogenic potential of DC within the uterus. In support of this notion, it has been shown that DC derived signals (particularly IL-12) promote IL-10 production by uterine NK cells [15], [36], which has been suggested to function as a negative feedback loop protecting from local excessive inflammation that would challenge pregnancy.

Uterine DC are currently considered an important cell subset for the regulation of decidual vascular development, as demonstrated by the impaired expansion of the decidual vascular bed together with decreased vessel permeability and blood flow observed on DC depleted implantation sites [6], [7]. DC were suggested to fine-tune decidual angiogenesis by controlling VEGF bioavailability [6], which is consistent with our results showing that serum concentrations of sFlt-1 were significantly reduced upon expansion of DC. However, these findings argue against the notion that DC themselves are a major source of this soluble antagonist [6], since in this case FL treatment would have been expected to increase sFlt-1 concentrations. Considering that most of the sFlt-1 released to the maternal circulation during pregnancy is derived from the trophoblast [37], and that DC depletion has been found to interfere with the differentiation of mouse trophoblast giant cells [7], it is possible that the decreased local levels of sFlt-1 reported by Plaks *et al.* upon DC depletion would result from an enhanced release to the circulation due to impaired trophoblast functions, as observed during the course of preeclampsia [38]. Importantly, our results further showed that the down-regulation of sFlt-1 levels upon DC expansion was attenuated in ↑DCØNK female mice. Together with the increased levels of PF4 expression, these findings suggest that NK cell depletion interferes with the local pro-angiogenic milieu induced during early pregnancy. This is in agreement with reports showing a decreased expression of VEGFs in the mesometrial decidua of NK cell depleted rat implantation sites [20], as well as recent studies demonstrating constitutive VEGF-A secretion by DBA^+^ mouse uNK cells [39]. Decidual angiogenesis appears to be controlled by the concerted actions of the VEGF and angiopoietin systems. A predominance of Angpt1 maintains vessel stability and integrity under steady-state conditions, whereas a switch towards low Angpt1/high Angpt2 promotes vessel destabilization rendering endothelial cells plastic and vulnerable to pro-angiogenic and remodeling factors such as VEGF [40]. Since the inhibitory effect on vessel integrity mediated by Angpt2 is an important pre-requisite both for angiogenesis and for vessel remodeling, the dysregulated expression of this factor observed in ↑DCØNK mice is consistent with the classical role ascribed to NK cells in promoting pregnancy-associated spiral artery transformation. Furthermore, since at least in humans uNK cells are known to be a source of Angpt2 expression [41], it cannot be ruled out that such an increase would result from NK cells themselves (which at gd 7.5 are partially reconstituted from precursors following anti-asialo GM1 treatment) in order to sustain normal arterial remodeling. However, it must also be noted that the vessel destabilizing effect of Angpt2 per se is insufficient to promote vessel outgrowth in the absence of stimulatory factors such as VEGF [42]. Thus, increased Angpt2 expression in the context of a strong expression of the anti-angiogenic PF4 as observed in ↑DCØNK mice may ultimately result in regression of destabilized vessels and compromise decidual vascularization. The corollary to these observations is that decidual vascular responses during early pregnancy appear to be dependent on the concerted actions of DC and NK cells, by virtue of their effect as modulators of the VEGF and angiopoietin pathways respectively.

Besides interfering with angiogenesis, the strong expression of PF4 observed in ↑DCØNK implantation sites further emphasizes the notion that pregnancy arrest in these mice is related to a local exacerbation of inflammation, as this chemokine has been described as a potent stimulator of neutrophil recruitment and activation [43]. This is consistent with our microarray results showing for instance an increased expression of Cxcl10, which similarly to PF4 exerts a potent pro-inflammatory and anti-angiogenic action that has been involved in the pathophysiology of preeclampsia [44]. Additionally, the gene expression data provides evidence that increased inflammation would result as a consequence of dysregulated DC functions in ↑DCØNK mice. As an example, spp1 is known to enhance DC maturation toward a Th1-promoting phenotype [45], and production of this cytokine by DC has been associated with the induction of Th17 responses [46]. Similarly, DC activated via dectin-1 (Clec7a) are also potent inducers of Th17 cells and cytotoxic CD8 responses [47], [48]. Thus, FL treatment appears to promote an immunogenic activation of DC in the uterus that severely compromises pregnancy progression in the absence of NK cells, highlighting a novel and important role played by these cells in the modulation of DC functions. Interestingly, recent studies have shown an association between preeclampsia and increased decidual densities of DC [49]. In this context, the results reported here highlight the relevance of the immunoregulatory interactions between DC and NK cells during early pregnancy that could be helpful to the understanding of the pathogenesis of reproductive disorders.

## Supporting Information

Table S1 **Sequences of qPCR primers used in the present study.**
(DOCX)

Table S2 **Statistical analysis at a false discovery rate (FDR) of 10% between ↑DCØNK and ↑DC implantation on gd 6.5. Results of SAM analysis for genes with q-values** ***<0.1*** **.**
(XLSX)

Table S3 **Selected gene sets enriched towards upregulated genes in ↑DCØNK found by GSEA.**
(XLSX)

Text S1 **Additional information of the materials and method section.**
(DOCX)
