# Correction: Uterine NK Cells Are Critical in Shaping DC Immunogenic Functions Compatible with Pregnancy Progression

**DOI:** 10.1371/annotation/9c332046-387a-4bbc-9549-2bcff73afd9a

**Published:** 2012-10-29

**Authors:** Irene Tirado-González, Gabriela Barrientos, Nancy Freitag, Teresa Otto, Victor L. J. L. Thijssen, Petra Moschansky, Petra von Kwiatkowski, Burghard F. Klapp, Elke Winterhager, Stefan Bauersachs, Sandra M. Blois

There is an error in the citation and copyright statement. The correct citation is: Tirado-González I, Barrientos G, Freitag N, Otto T, Thijssen VLJL, et al. (2012) Uterine NK Cells Are Critical in Shaping DC Immunogenic Functions Compatible with Pregnancy Progression. PLoS ONE 7(10): e46755. doi:10.1371/journal.pone.0046755

The correct copyright is: © Tirado-González et al. This is an open-access article distributed under the terms of the Creative Commons Attribution License, which permits unrestricted use, distribution, and reproduction in any medium, provided the original author and source are credited. 

