# Supplementary material for: Uterine NK Cells Are Critical in Shaping DC Immunogenic Functions Compatible with Pregnancy Progression
Source: PLoS One. 2012 Oct 8;7(10):e46755. doi: 10.1371/journal.pone.0046755 (PMC3466312; doi:10.1371/journal.pone.0046755)
Supplement: Table S1 — Sequences of qPCR primers used in the present study. (DOCX) [file pone.0046755.s001.docx]

**Table S1.** Sequences of qPCR primers used in the present study.

| **Gene** | **Forward primer (5’-3’)** | **Reverse primer (5’-3’)** |
| --- | --- | --- |
| IL-11 | ATTTTGGTACTTGGAGGGGG | CATAGAGACCCCAGAACCCA |
| Angpt1 | TGCATTCTTCGCTGCCATTCT | ATTGCCCATGTTGAATCCGGT |
| Angpt-2 | TCCAAGAGCTCGGTTGCTAT | AGTTGGGGAAGGTCAGTGTG |
| Tek | ATGTGGAAGTCGAGAGGCGAT | CCTGAACCTTATACCGGATGA |
| PF4 | CCAGCCTGGAGGTGATCAA | GGCAAATTTTCCTCCCATTCT |
| Cxcr2 | agcaaacacctctactaccctcta | gggctgcatcaattcaaatacca |
| Ptpn5 | GGCATCTCTTTCTGCTTGCT | AACCCTGGACATGTGCTGTAG |
| Irf7 | AGCATTGCTGAGGCTCACTT | TGATCCGCATAAGGTGTACG |
| Sirpb1 | CCCGTTCACAGGAGAACATT | CCGGAGACCATAGGTGAAGA |
| Spp1 | TCTGATGAGACCGTCACTGC | AGGTCCTCATCTGTGGCATC |
| HPRT | GTTGGATACAGGCCAGACTTTGT | CACAGGACTAGAACACCTGC |
