# Supplementary material for: Uterine NK Cells Are Critical in Shaping DC Immunogenic Functions Compatible with Pregnancy Progression
Source: PLoS One. 2012 Oct 8;7(10):e46755. doi: 10.1371/journal.pone.0046755 (PMC3466312; doi:10.1371/journal.pone.0046755)
Supplement: Text S1 — Additional information of the materials and method section. (DOCX) [file pone.0046755.s004.docx]

**Text S1.** Additional information of the materials and method section.

***Phosphorylated histone H3 (PHH3) and progesterone receptor (Prg) staining***. Staining of 8 μm sections was performed by washing in TBS, followed by blocking of endogenous peroxidase through incubation with 3% H_2_O_2_ in methanol for 30 min at room temperature. After incubation with 2% normal serum for 20 min, primary Ab against PHH3 (1:100, sc-8656-R) or Prg were incubated O.N. at 4°C. The slides were then washed and incubated with HRP conjugated secondary Ab (Jackson ImmunoResearch) for 1h at room temperature. The signal was detected by incubating at room temperature with a 0.2 mg/ml diaminobenzidine (Dako Cytomation) 0.05% H_2_O_2_ substrate solution. After washing, nuclei were counterstained with 0.1% Mayer’s hematoxylin followed by a standard dehydration procedure and mounting in Vitro-Clud medium (R. Langenbrinck). Negative controls with irrelevant IgG showed no specific immunoreactivity. Tissue sections were examined using a light microscope (Axiophot) and photographs taken with Axio Cam HRc. Photo documentation was performed using the digital image analysis system Spot advanced software, version 8.6 (Visitron Systems).

***Histology and immunofluorescence***. For histological analysis, uterine sections from gd 5.5 and 6.5 were fixed with 10% buffered formalin, dehydrated in ethanol, embedded with paraffin, and stained with haematoxylin-eosin. For immunofluorescence study, serial sections were prepared at 8μm. Multiple implantation sites (2-3) from multiple females (4-7) were stained at gd 5.5 and 6.5 after our standard protocol. Briefly, slides were washed 3 times in TBS for 5 min, blocked with 2% normal serum for 20 min and incubated o.n. at 4°C with the primary antibody against connexin 43 (Cx-43) (1:100, sc-9059). Negative controls were established by replacing the primary Ab with irrelevant IgG. After washing, Cx-43 stained sections were incubated 1h at RT with FITC-conjugate secondary antibody (Jackson ImmunoResearch). Nuclei in all sections were counterstained by incubating 5 min in DAPI solution, followed by washing and mounting in Shandon Immu-Mount^TM^ (Thermo Scientific). Sections were analyzed using a confocal laser scanning microscope (cLSM 510, Carl Zeiss).

***Real Time qPCR.*** Total RNA was extracted from implantation site tissues on gd 5.5 and 6.5 using the Nucleospin RNA/protein isolation kit (Macherey-Nagel, Germany). After DNase digestion (Invitrogen, Germany), cDNA was generated using random primers (Invitrogen) followed by quantitative real-time RT-PCR performed on the TaqMan 7500 System (Applied Biosystems). For each reaction, 1 µL cDNA, synthesized from 1 µg RNA in 25 µL, was used in a total volume of 12 µL containing 6.25µL of Power SYBR Green PCR mastermix (Applied Biosystems), 3.75 µL DEPC water and 450 nM of the appropriate forward and reverse primer. Primers used in PCRs are shown in Table S1. The PCR profile was as follows: 2 minutes at 50°C, 10 minutes at 95°C, followed by 40 cycles of 15 seconds at 95°C and 60 seconds at 60°C. Subsequently, a melting curve analysis was performed which consisted of 70 cycles of 10 seconds with a temperature increment of 0.5°C/cycle starting at 60°C. The relative expression was calculated according to the equation Rel. Exp (RE) = 2-DDCt. The obtained Ct value of each gene of interest was normalized to the Ct of the reference genes as follows: Ct_norm_ = Ct_goi_ - Ct_ref_ with norm = normalized, goi = gene of interest, and ref = reference gene.

***Soluble fms-related tyrosine kinase 1 (Flt-1) ELISA.*** The quantification of Flt-1 serum levels was performed using the mouse Flt-1 Quantikine Immunoassay (R&D Systems, Cat MVR100) following the manufacturer’s recommendations. A standard curve was generated by incubating 50 µl of Flt-1 standard (at serial 2-fold dilutions ranging from 8000 to 125pg/ml) for 2 h at room temperature in a microtiter plate precoated with Flt-1 capture Ab (supplied within the kit). Mouse serum samples were assayed at ½ dilutions. The plates were then washed and incubated for 2 h with 100 µl/well of the mouse Flt-1 conjugate at room temperature. For the detection of the colorimetric reaction, 100 µl of TMB Substrate Solution were added to each well and incubated for 30 min followed by the addition of 100 μl/well 2N H2SO4. The OD was determined at 450 nm and the calculation of the results was performed as described above.
